# Warming Alters Expressions of Microbial Functional Genes Important to Ecosystem Functioning

**DOI:** 10.3389/fmicb.2016.00668

**Published:** 2016-05-06

**Authors:** Kai Xue, Jianping Xie, Aifen Zhou, Feifei Liu, Dejun Li, Liyou Wu, Ye Deng, Zhili He, Joy D. Van Nostrand, Yiqi Luo, Jizhong Zhou

**Affiliations:** ^1^State Key Joint Laboratory of Environment Simulation and Pollution Control, School of Environment, Tsinghua UniversityBeijing, China; ^2^Institute for Environmental Genomics, University of Oklahoma, NormanOK, USA; ^3^Department of Microbiology and Plant Biology, University of Oklahoma, NormanOK, USA; ^4^School of Mineral Processing and Bioengineering, Central South UniversityChangsha, China; ^5^CAS Key Laboratory of Environmental Biotechnology, Research Center for Eco-Environmental Sciences, Chinese Academy of SciencesBeijing, China; ^6^Earth Science Division, Lawrence Berkeley National Laboratory, BerkeleyCA, USA

**Keywords:** RNA, functional gene expression, global climate change, warming, GeoChip

## Abstract

Soil microbial communities play critical roles in ecosystem functioning and are likely altered by climate warming. However, so far, little is known about effects of warming on microbial functional gene expressions. Here, we applied functional gene array (GeoChip 3.0) to analyze cDNA reversely transcribed from total RNA to assess expressed functional genes in active soil microbial communities after nine years of experimental warming in a tallgrass prairie. Our results showed that warming significantly altered the community wide gene expressions. Specifically, expressed genes for degrading more recalcitrant carbon were stimulated by warming, likely linked to the plant community shift toward more C_4_ species under warming and to decrease the long-term soil carbon stability. In addition, warming changed expressed genes in labile C degradation and N cycling in different directions (increase and decrease), possibly reflecting the dynamics of labile C and available N pools during sampling. However, the average abundances of expressed genes in phosphorus and sulfur cycling were all increased by warming, implying a stable trend of accelerated P and S processes which might be a mechanism to sustain higher plant growth. Furthermore, the expressed gene composition was closely related to both dynamic (e.g., soil moisture) and stable environmental attributes (e.g., C_4_ leaf C or N content), indicating that RNA analyses could also capture certain stable trends in the long-term treatment. Overall, this study revealed the importance of elucidating functional gene expressions of soil microbial community in enhancing our understanding of ecosystem responses to warming.

## Introduction

Global climate change resulting from anthropogenic activities (Novotny et al., 2007) has become one of the greatest scientific and political concerns (IPCC, 2007). Near the summit of the Mauna Loa volcano in Hawaii, the daily concentration of atmospheric CO_2_ has reached a worrisome milestone, 400 ppm, in Monastersky (2013). The yearly average global CO_2_ concentration is predicted to surpass 400 ppm in only a few years (Monastersky, 2013). The emissions of CO_2_ and other greenhouse gasses had driven the Earth’s average temperature to increase by 0.74°C in the 20th century, which may continue to increase by 1.1–6.4°C at the end of this century (IPCC, 2007).

Within the global climate change context, the soil microbial community is likely to be influenced by the atmospheric warming. The temperature increase itself affects almost all chemical and biological processes (Shaver et al., 2000) and thus may alter the soil microbial community directly. Meanwhile, warming may influence the soil microbial community via its indirect effects on plant communities (Cheng et al., 2010). For example, plant community shift toward more C_4_ species was observed in the warming treatment plots (An et al., 2005; Wan et al., 2005; Zhang et al., 2005; Luo et al., 2009). C_3_ and C_4_ species differ in their photosynthesis pathways to fix and reduce inorganic CO_2_ into organic compounds, and C_4_ species were generally believed to have lower plant tissue quality, e.g., higher lignin content and greater C:N ratio (Kephart and Buxton, 1993). Thus, the above-ground plant community shift under warming may lead to changes in plant-derived soil carbon (C) input that is important substrate for soil microbial community, and hence may alter soil microbial community.

Influences of warming on soil microbial communities have been assessed by various molecular approaches such as phospholipid fatty acid (PLFA) analysis (Zogg et al., 1997; Zhang et al., 2005, 2011; Rinnan et al., 2007; Frey et al., 2008), Biolog (Rinnan et al., 2007; Zhang et al., 2011) or a series of DNA-based methods, including traditional molecular tools [e.g., terminal restriction fragment length polymorphism (T-RFLP), denaturing gradient gel electrophoresis (DGGE), etc.] (Deslippe et al., 2005, 2012; Cookson et al., 2007; Schindlbacher et al., 2011; Sheik et al., 2011; Stockdale et al., 2013) and newly emerged metagenomic technologies (e.g., high throughput sequencing and microarrays; Mackelprang et al., 2011; Sheik et al., 2011; Hayden et al., 2012; Kuffner et al., 2012; Yergeau et al., 2012). Substantial influences of warming on soil microbial communities were found in the majority of these studies, from aspects of fingerprint, taxonomic and phylogenetic compositions. Moreover, crucial roles of soil microbial community in regulating ecosystem C dynamics under warming were discovered by hybridizing DNA from soil microbial community on GeoChip3.0 (Zhou et al., 2012), a functional gene array-based metagenomic tool that can allow to assess genes in a highly comprehensive and standardized manner.

Due to the importance of microbial communities in mediating soil biogeochemical processes (van Bruggen and Semenov, 2000; Jackson et al., 2003; Xue et al., 2011), influences of warming on microbial community activities may potentially affect the biogeochemical cycling and ecosystem functioning. However, DNA-based technologies are hard to directly reflect the actual activity of active microbial populations (McGrath et al., 2010; Simon and Daniel, 2011; Franzosa et al., 2015). In contrast, RNA-based approaches can provide better insights into functional attributes of active populations in microbial community (McGrath et al., 2010; Simon and Daniel, 2011; Takasaki et al., 2013). RNA-based approaches have already been applied in global climate change studies, e.g., discovering the microbial response to ocean acidification due to increased atmospheric CO_2_ concentration (Gilbert et al., 2008) and some investigations in peat soils (Tveit et al., 2013; Tuorto et al., 2014).

By using DNA-based GeoChip3.0, we discovered crucial roles of soil microbial community in regulating ecosystem C dynamics under warming through three mechanisms: shifts in the functional gene composition of soil microbial community, enriched genes for labile C but not recalcitrant C degradation, and enriched genes for nutrient cycling (Zhou et al., 2012). To better understand functional attributes of active populations in microbial community under warming, we used RNA-based GeoChip 3.0 in this study to assess effects of warming on functional gene expressions of soil microbial community in the same experimental site after 9 years of warming (targeting 2°C above ambient temperature) at the Great Plain Apiaries in Oklahoma, USA. GeoChip 3.0 contains approximately 28, 000 probes covering more than 57,000 gene variants from 292 functional gene families involved in C, nitrogen (N), phosphorus (P) and sulfur (S) cycles and other processes (He et al., 2010). We hypothesized that expressions of key functional genes of soil microbial community involved in important biochemistry processes would be stimulated by warming, and these changes would be closely linked to the dynamics of environmental attributes. Our results showed that expressions of genes involved in recalcitrant C degradation, P and S cycling were stimulated by warming, but both increased and decreased gene expressions were observed for labile C degradation and N cycling. Moreover, the expressed gene composition was closely related to both dynamic (e.g., soil moisture) and stable environmental attributes (e.g., C_4_ leaf C or N content).

## Materials and Methods

### Study Site

This study was conducted at the Kessler Farm Field Laboratory (KFFL) located in the Great Plain Apiaries in McClain County, Oklahoma, USA (34°58′54′′N, 97°31′14′′W; Luo et al., 2001). The grassland is dominated by C_4_ grasses (*Andropogon gerardii, Sorghastrum nutans, Schizachyrium scoparium, Panicum virgatum, and Eragrostis* spp.), C_3_ forbs (*Ambrosia psilostachya and Xanthocephalum texanum*), and C_3_ annual grass (*Bromus japonicus*; Zhang et al., 2005; Sherry et al., 2008). Based on Oklahoma Climatological Survey, the mean annual temperature and precipitation in this site was 16.3°C and 967 mm, respectively. The soil is silt loam (36% sand, 55% silt, and 10% clay in the top 15 cm) and part of Nash–Lucien complex, typically with high fertility, neutral pH, high available water capacity and a deep moderately penetrable root zone (USDA, 1963).

The experiment was established in November 1999 with a blocked split-plot design, in which warming is a primary factor nested by clipping (annual removal of above-ground biomass). In this study, we focus on examining the effects of warming on expressions of functional genes in soil microbial community and thus only samples from unclipped sub-plots were collected. There were six replicates for each treatment. In 1 m × 1 m subplots, the warming treatment (targeting +2°C) was created by suspending infrared radiators (Kalglo Electronics, Bethlehem, Pennsylvania) 1.5 m above the ground; while dummy infrared radiators were used in control plots to exclude the shading effect of the device itself. Soil samples were collected from the top 15 cm layer in October 2008. In each subplot, four soil cores (2.5 cm diameter × 15 cm deep) were taken, likely including both rhizosphere soil and bulk soil, and then composited to one sample. Like many long term experimental sites, destructive sampling is restricted. Thus, only one-time point can be allowed for sampling. All samples were sieved by 2 mm sieves and stored at -80°C before analyses.

The measurements or calculations for environmental attributes in 2008, including soil temperature, NH_4_^+^, NO_3_^-^ and total N contents, as well as heterotrophic respiration and autotrophic respiration, were described in detail in Supplementary Materials and Methods. Other environmental attributes used for CCA and Mantel test (see Statistical Analysis) were from previous studies to investigate treatment effects (Niu et al., 2010; Cheng et al., 2011; Xu et al., 2012a,b, 2014, 2015; Zhou et al., 2012), all collected in 2008 (Supplementary Table S1).

### Microbial Community Total RNA Isolation and Labeling

Total DNA and RNA from the soil microbial community were extracted from 5 g soil according to the method developed previously (Hurt et al., 2001). Total RNA was isolated and purified with Qiagen RNeasy^®^Mini Kit (Qiagen, Germantown, MD, USA). RNA quantity and quality were assessed based on the ratios of 260/280 nm and 260/230 nm absorbance by a NanoDrop ND-1000 Spectrophotometer (NanoDrop Technologies Inc., Wilmington, DE, USA), as well as agarose gel image.

Total RNA (500 ng) was amplified with modified protocols described previously (Gao et al., 2007) since limited amount of total RNA was obtained from soil samples. Briefly, first strand cDNA was synthesized with random primer T7N6 bearing a T7 promoter and six random oligonucleotides and reverse transcriptase superscript III. The product was used for the second strand cDNA synthesis with a cocktail containing *Escherichia coli* DNA polymerase I, RNaseH, and DNA ligase. After the synthesis of second strand cDNA, T4 DNA polymerase was added to polish the end. The double strand DNA was then purified with phenol:chloroform:isoamyl alcohol and *in vitro* transcribed with MegaScript T7 kit (Life technologies, Grand Island, NY, USA). The amplified RNA was purified with Qiagen RNeasy Mini Kit (Qiagen, Germantown, MD, USA) and eluted in RNase free H_2_O. Amplified RNA was labeled with fluorescent dye Cy5 via reverse transcription reaction. The 10 μg amplified RNA was mixed with 3.3 μl random primers (3.0 μg/μl; Life Technologies, Grand Island, NY, USA) and adjusted the volume to 10 μl with water. After 10 min of denaturation at 70°C and immediate chilling on ice, the labeling mix containing 6 μl 5X first strand buffer, 3 μl 0.1 M DTT, 1.5 μl dNTP mix (10 mM dA/G/CTP and 0.5 mM dTTP), 1 μl RNase inhibitor and 1 μl Cy5-dUTP was added and incubated in the dark at room temperature for 10 min. Then 1 μl Superscript reverse transcriptase II was added to the mixture and incubated at 42°C for 2 h. Labeled products were purified with QIAquick PCR purification kit (Qiagen, Germantown, MD, USA) and vacuum dried.

### GeoChip Hybridization and Imaging Processing

GeoChip 3.0 was used in this study as described previously (Wu et al., 2006; Xue et al., 2013). The Cy5-labeled cDNA was re-suspended in 50 μl hybridization solution [40% formamide, 5x SSC, 5 μg of unlabeled herring sperm DNA (Promega, Madison, WI, USA), and 0.1% SDS] and 2 μl universal standard DNA (0.2 pmol μl^-1^) labeled with the fluorescent dye Cy-3 (Liang et al., 2010), denatured at 95°C for 3 min and maintained at 65°C until loaded onto microarray slides. Arrays were hybridized on a MAUI Hybridization Station (Roche, South San Francisco, CA, USA) for 10 h at 42°C. After washing and drying, the microarrays were scanned using a ScanArray Express Microarray Scanner (Perkin Elmer, Boston, MA, USA) at 633 nm with a laser power of 90% and a photomultiplier tube (PMT) gain of 75%. The obtained images were analyzed by ImaGene version 6.0 (Biodiscovery, El Segundo, CA, USA) to determine the intensity of each spot.

Raw data from ImaGene were submitted to the Microarray Data Manager System^1^ and analyzed by the following steps: (i) spots (probes) flagged as 1 or 3 by Imagene and with a signal to noise ratio (SNR) less than 3.0 were removed as poor-quality spots; (ii) Cy5 intensities of functional genes were normalized by a two-step method based on mean Cy3 intensities of universal standards in each sub-grid within a slide and in each slide among samples; (iii) outliers were defined as probe intensities out of the 95% confident interval for each probe in control or warming treatment and removed until no outliers were identified; (iv) genes with less than 0.34 time of the final positive probes or two probes were removed; (v) probes that appeared in less than two of six replicates in each treatment were removed. After these steps, relative abundances were calculated through dividing signal intensities for individual probes by the sum of intensities for all probes detected in each sample. Then relative abundances were multiplied by the mean value of intensity sums over all samples. A natural logarithm transformation (x+1) was performed for each amplified relative abundance plus 1.

### Statistical Analysis

Detrended correspondence analysis (DCA), three non-parametric tests (multiple response permutation procedure, MRPP; permutational multivariate analysis of variance, ADONIS; analysis of similarity, ANOSIM), Mantel test and canonical correlation analysis (CCA) were performed by R version 2.9.1 (The R Foundation for Statistical Computing, Vienna, Austria). The three non-parametric tests were based on *Bray*–*Curtis, Horn*, and *Euclidean* dissimilarity indexes; whilst mantel test were based on *Euclidean* dissimilarity. To construct a CCA model, 11 environmental attributes were selected based on their biological importance and *p*-values of single-factor CCA models, including soil temperature, soil moisture, total soil organic C (TOC), the proportion of soil C derived from C_4_ plant species (F-C_4_), soil ^15^N content, C_3_ species above-ground biomass, C_4_ species above-ground biomass, C_4_ leaf C content, C_4_ leaf C:N ratio, C_3_ leaf C content, and below net primary productivity (BNPP). Then the automatic forward selection by permutation tests was applied in the stepwise analysis to choose constrained variables in the final CCA model, combined with the variance inflation factor (VIF) criterion (VIF < 20).

Treatment differences between warming and control for expressed gene abundances were analyzed by two-tailed paired *t*-test by R version 2.9.1 (The R Foundation for Statistical Computing, Vienna, Austria). The significance was defined as *p* ≤ 0.05, while marginally significance was defined as 0.05 < *p* ≤ 0.10.

## Results

### Effects of Warming on Composition of Expressed Functional Genes in Soil Microbial Community

**FIGURE 1:**
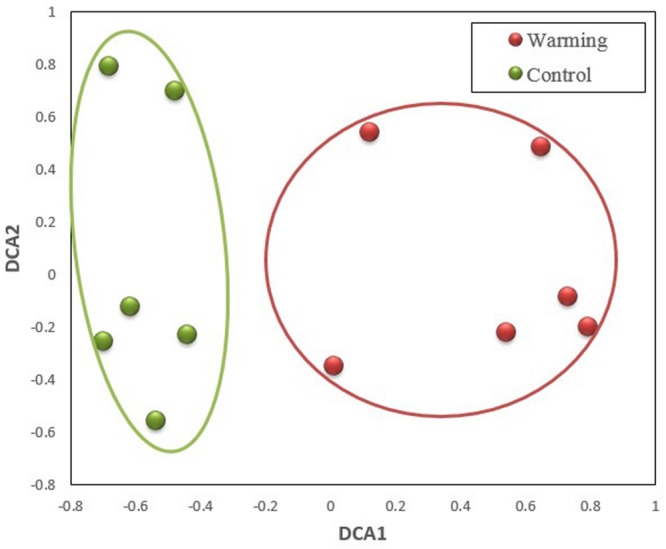
**Detrended correspondence analysis (DCA) of microbial community composition in control and warming treatments based on expressed functional genes**.

The GeoChip analysis was performed with cDNA that was reversely transcribed from total RNA to investigate functional gene expressions. A total of 2,347 probes (∼10% of the probes on the arrays) were detected in all samples, of which 1,733 and 1,947 were in control and warming treatments, respectively. In this way, there were a big portion, 1333 probes (56.8%), were overlapped between two treatments. Meanwhile, among all 2,347 detected probes, 400 (17.0%) and 414 (26.2%) were unique to control and warming treatments, respectively. The composition of expressed genes differed significantly (*p* ≤ 0.005) between control and warming, as revealed by all three non-parametric dissimilarity tests (MRPP, ADONIS, and ANOSIM) based on *Bray–Curtis* dissimilarity (**Table 1**). Consistently, DCA profile also illustrated that the warming samples were separated clearly from control samples (**Figure 1**).

**Table 1:** Non-parametric analyses to test dissimilarity of communities between warming and control.

Warming vs. Control	ADONIS^a^		ANOSIM^b^		MRPP^c^	
	*F*	*P*^d^	*R*	*P*	δ	*P*
*Bray*–*Curtis*	2.874	0.005	0.343	0.005	0.375	0.003
*Horn*	2.951	0.004	0.335	0.009	0.353	0.005
*Euclidean*	2.293	0.002	0.480	0.004	273.005	0.003

*^a^Permutational multivariate analysis of variance using distance matrices. Significance tests were performed by *F*-tests based on sequential sums of squares from permutations of the raw data. ^b^Analysis of similarities. Statistic *R* is based on the difference of mean ranks between groups and within groups. The significance of observed *R* is assessed by permuting the grouping vector to obtain the empirical distribution of *R* under null-model. ^c^Multi-response permutation procedure. Statistic δ is the overall weighted mean of within-group means of the pairwise dissimilarities among sampling units. The significance test is the fraction of permuted deltas that are less than the observed delta. ^d^*p*-value of corresponding significance test. All three tests are multivariate analyses based on *Bray–Curtis, Horn*, and *Euclidean* dissimilarity indexes.*

### Effects of Warming on Expressed Functional Genes Involved in Biochemical Cycling

**FIGURE 2:**
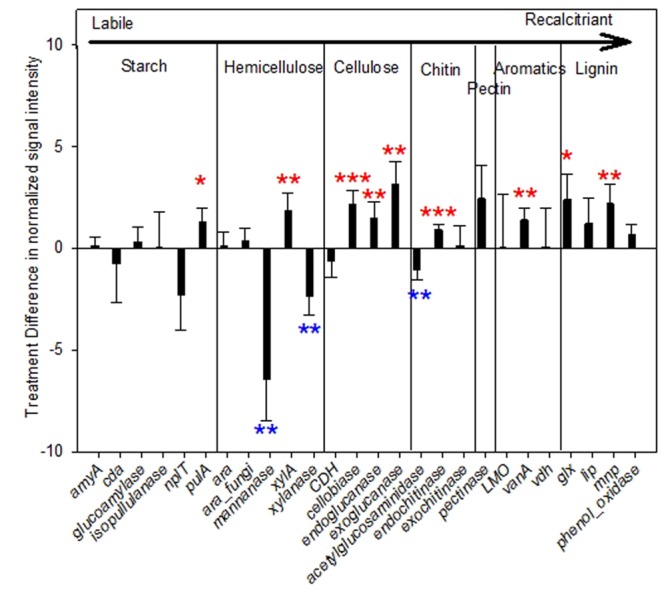
**Difference of normalized signal intensity between warming and control for expressed functional genes involved in carbon (C) degradation.** The complexity of carbon is presented in order from labile to recalcitrant from left to right. Error bars represent standard error. Significance according to two tailed paired *t*-test were labeled by ^∗∗∗^*p* ≤ 0.01, ^∗∗^*p* ≤ 0.05, ^∗^*p* ≤ 0.10; while red and blue colors represent increases and decreases caused by warming.

Expressed genes involved in biochemical cycles of C/N/P/S are of our particular interests. A total of 26 expressed genes encoding enzymes for degrading C components were detected. Among them, 34.6% (9 of 26) had significantly or marginally significantly higher abundances in warming than control samples (**Figure 2**), including not only labile, but also recalcitrant C decomposition genes, e.g., *vanA* encoding vanillate *O*-demethylase oxygenase (*p* = 0.02) for degrading aromatic compounds; *glx* encoding glyoxal oxidase (*p* = 0.07) and *mnp* encoding manganese peroxidase (*p* = 0.03) for lignin degradation. Meanwhile, abundances of three expressed genes were decreased by warming significantly, all involved in labile C degradation, including those encoding mannanase (*p* = 0.03) and xylanase (*p* = 0.02) for hemicellulose degradation, and acetylglucosaminidase (*p* = 0.04) for chitin degradation. The rest of C degradation genes did not differ significantly by warming.

**FIGURE 3:**
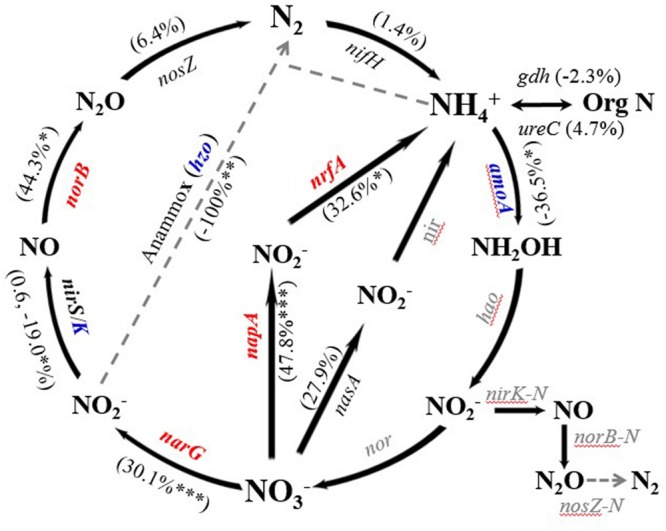
**Percentage change of normalized signal intensity from expressed functional genes involved in nitrogen (N) cycling to warming.** Significance according to two tailed paired *t*-test were labeled by ^∗∗∗^*p* ≤ 0.01, ^∗∗^*p* ≤ 0.05, ^∗^*p* ≤ 0.10. Red and blue colors represent increases and decreases caused by warming; while gray color means that the expressed genes were not present on the version of GeoChip used, or were undetected.

There were 13 expressed genes detected in N cycling. Among them, 30.7% (4 of 13) were stimulated significantly or marginally significantly by warming (**Figure 3**). On the contrary, warming also decreased abundances of three expressed genes significantly or marginally significantly, including *hzo* encoding hydrazine oxidase (*p* = 0.03) for anammox, *nirK* encoding copper-containing nitrite reductase (*p* = 0.06) for denitrification, and *amoA* encoding ammonia monooxygenase (*p* = 0.06) for nitrification. Abundances of the rest of expressed N genes did not differ between control and warming, including those for ammonification, assimilatory N reduction and N fixation processes.

**FIGURE 4:**
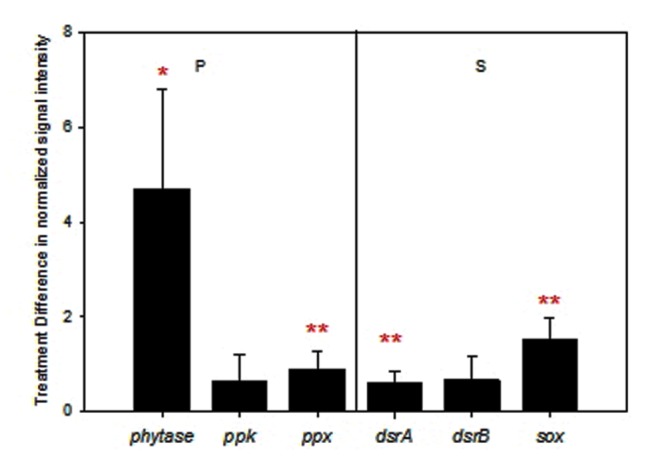
**Difference of normalized signal intensity between warming and control for expressed functional genes involved in phosphorus (P) and sulfur (S) cycling.** Error bars represent standard error. Significance according to two tailed paired *t*-test were labeled by ^∗∗∗^*p* ≤ 0.01, ^∗∗^*p* ≤ 0.05, ^∗^*p* ≤ 0.10; while red and blue colors represent increases and decreases caused by warming.

Different from C and N cycles, all expressed genes involved in P and S cycles were stimulated by warming on average (**Figure 4**). Among them, changes in *ppx* encoding exopolyphosphatase (*p* = 0.02) and the gene encoding phytase (*p* = 0.08) for P utilization, *dsrA* encoding the large subunit of sulfite reductase (*p* = 0.02) for sulfite reduction, and *sox* for sulfur oxidation (*p* < 0.001) were significant or marginally significant.

### Linkage between Environmental Attributes and Expressed Gene Structure

**FIGURE 5:**
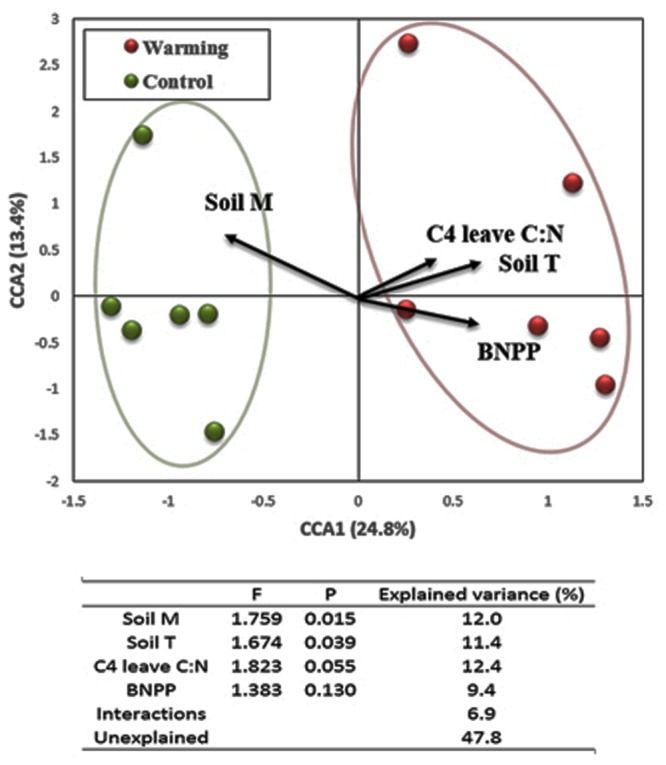
**Canonical correspondence analysis (CCA) of microbial community composition based on expressed functional genes with selected environmental variables.** The table presents results from partial CCA and variation partitioning analysis (VPA). The selected environmental variables include soil moisture (Soil M), soil temperature (Soil T), leaf C:N ratio in C_4_ species (C_4_ leaf C:N), and below-ground net primary productivity (BNPP).

Canonical correlation analysis was performed to investigate the relationship between environmental attributes and the structure of expressed functional genes in microbial communities. Four attributes were chosen in the final CCA model (*F* = 1.91, *p* = 0.005), including soil temperature (*F* = 1.76, *p* = 0.015 for partial CCA), soil moisture (*F* = 1.67, *p* = 0.039 for partial CCA), C_4_ leaf C:N (*F* = 1.82, *p* = 0.055 for partial CCA) and BNPP (*F* = 1.38, *p* = 0.130 for partial CCA). In the CCA profile (**Figure 5**), warming samples separated clearly from control samples along the first canonical axis (CCA1), explaining 24.8% of the total variation in expressed gene composition. Projections of environmental attributes by CCA revealed that warming samples were positively correlated with soil temperature, BNPP and C_4_ leaf C:N, but negatively correlated with soil moisture. These projections were consistent with observations in these attributes when investigating treatment effects (Niu et al., 2010; Xu et al., 2012a, 2014). Moreover, variation portioning analysis (VPA) was performed based on partial CCA and found that each single factor explained 9.4–12.4% of the total variance in expressed functional gene structure; while their interaction explained 6.9% in total, leaving 47.8% as unexplained (**Figure 5**).

To further examine the linkage between environmental attributes and certain groups of expressed genes, Mantel test was performed (**Table 2**). The structure of all expressed C degradation genes was correlated significantly (*p* ≤ 0.05) with soil moisture, TOC, above-ground net primary productivity (ANPP) and C_4_ species leaf C content. Other than these variables, the proportion of recalcitrant C pool in TOC, C_4_ AGB and BNPP were correlated significantly (*p* < 0.05) with expressed genes for decomposing recalcitrant C components only (e.g., aromatic compounds). In N cycling, surprisingly, no significant correlation was observed between any expressed gene groups and soil NH_4_^+^, NO_3_^-^ or total N content (**Table 2**). Rather, the structure of all expressed N genes was correlated significantly with C_4_ species leaf N content (*p* = 0.03) and its C:N ratio (*p* = 0.02). Moreover, expressed assimilatory N reduction genes were correlated with TOC significantly (*p* = 0.04); while expressed ammonification genes were correlated significantly with soil ^13^C content (*p* = 0.02) and the proportion of soil C derived from C_4_ species (*p* = 0.03).

**Table 2:** Mantel tests between composition of expressed gene catalogs involved in C degradation/N processes and environmental variables.

	M	RP	TOC	^13^C	F-C_4_	C_4_ AGB	ANPP	BNPP	C_4_L-C	C_4_L-N	C_4_L-C:N
C degradation	^∗∗^		^∗∗^				^∗∗^		^∗∗^		
Starch	^∗∗^										
Hemicellulose	^∗∗∗^								^∗∗^		
Cellulose									^∗∗^		
Chitin	^∗∗^						^∗∗^				
Pectin		^∗∗^	^∗∗∗^				^∗∗∗^	^∗∗^	^∗∗^		
Aromatics		^∗∗^	^∗∗^			^∗∗^		^∗∗^			
Lignin	^∗∗^		^∗∗^				^∗∗∗^	^∗∗^	^∗∗^		
N										^∗∗^	^∗∗^
Ammonification				^∗∗^	^∗∗^						
Anammox											
Assimilatory N reduction	^∗∗^		^∗∗^					^∗∗^			
Denitrification	^∗∗^									^∗∗^	^∗∗^
Dissimilatory N reduction								^∗∗∗^			
Nitrification											
Nitrogen fixation										^∗∗^	^∗∗^

*The significance was labeled as ^∗∗∗^*p* ≤ 0.01, ^∗∗^*p* ≤ 0.05. Significant correlations were observed between expressed gene compositions and soil moisture (M), recalcitrant C pool (RP), total organic C (TOC), ^*13*^C content, the proportion of soil C derived from C_*4*_ plant species (F-C_*4*_), C_*4*_ species aboveground biomass (C_*4*_ AGB), aboveground net primary productivity (ANPP), belowground net primary productivity (BNPP), C_*4*_ leaf C content (C_*4*_L-C), C_*4*_ leaf N content (C_*4*_-N), and C_*4*_ leaf C:N (C_*4*_L-C:N). There were no significant correlations between expressed gene compositions and soil temperature (T), labile C pool 1 (LP1), labile C pool 2 (LP2), NH_*4*_^+^, NO_*3*_^-^, total N (TN), and ^*15*^N in soil, C_*3*_ species aboveground biomass (C_*3*_ AGB), C_*3*_ leaf C content (C_*3*_L-C), C_*3*_ leaf N content (C_*3*_-N), C_*3*_ leaf C:N (C_*3*_L-C:N), soil respiration (Soil R), heterotrophic respiration (HR), and autotrophic respiration (AR).*

## Discussion

One of the biggest scientific challenges of the 21st century is to better understand biological mechanisms regulating ecosystem responses to climate change and feedbacks that can amplify or dampen climate change (Heimann and Reichstein, 2008). Within this context, an understanding of soil microbial community is critical to our ability to assess terrestrial ecosystem responses and feedbacks (Bardgett et al., 2008). In the previous study conducted in the same experimental site, by using DNA-based GeoChip analysis, shifted functional gene composition of soil microbial community was observed under a long-term warming treatment (Zhou et al., 2012). In this study, by using RNA-based GeoChip analysis, we also found that the compositions of expressed functional genes in soil microbial community were altered significantly under warming. This finding well agrees with the general notion of shifted microbial community composition under warming observed in either the same (Zhang et al., 2005; Sheik et al., 2011) or different experiment sites (Zogg et al., 1997; Deslippe et al., 2005, 2012; Cookson et al., 2007; Rinnan et al., 2007; Frey et al., 2008; Mackelprang et al., 2011; Sheik et al., 2011; Zhang et al., 2011; Hayden et al., 2012; Kuffner et al., 2012; Yergeau et al., 2012; Stockdale et al., 2013), mainly tested by DNA-based methods.

In this experimental site, alterations in soil microclimate (e.g., soil temperature) were observed under warming (Xu et al., 2012b). The quantity of soil C input from plant that provides substrates for microorganisms likely increased under warming due to the total AGB increase. However, its quality possibly decreased as the proportion of soil C derived from C_4_ species increased with warming (Zhou et al., 2012), reflecting the plant community shift toward more C_4_ species with generally higher lignin content (Kephart and Buxton, 1993) and observed greater C:N ratio in plant tissues (Niu et al., 2010). These changes involved in soil physicochemical properties could be linked to the composition shift of expressed functional genes in soil microbial community. Close relationship between soil physicochemical properties and soil microbial community are widely recognized (Fierer et al., 2009; Peralta et al., 2013). Consistently, the CCA and VPA analyses revealed that the model consisting of soil temperature, soil moisture, leaf C:N ratio in C_4_ species and BNPP explained 52.2% of total variance in the structure of all expressed functional genes significantly (*p* = 0.005).

Different results in characterizing microbial communities by using DNA and RNA-based methods were observed in this and many other studies (Frias-Lopez et al., 2008; Gilbert et al., 2008; Baldrian et al., 2012; DeAngelis and Firestone, 2012). For example, in the previous study, unchanged recalcitrant C degradation were discovered under warming based on DNA-GeoChip analysis (Zhou et al., 2012), though the plant community shift towards more C_4_ species had long been observed in the field (An et al., 2005; Wan et al., 2005; Zhang et al., 2005; Luo et al., 2009). In this study, expressed genes for degrading more recalcitrant C components (i.e., aromatic components and lignin) increased with warming. Moreover, none of the functional genes involved in C degradation or N cycling decreased with warming based on the DNA-GeoChip analysis in the previous study (Zhou et al., 2012), while different changing directions (increase and decrease) were observed for expressed genes involved in labile C degradation and N cycling in this study. These phenomena were likely due to the fact that DNA and RNA-based methods characterize microbial communities in different aspects. RNA-based analyses only detect instantaneously expressed genes from metabolically active populations in the microbial community (Dell’Anno et al., 1998; Mills et al., 2005); while DNA-based surveys cannot discern active or inactive members if microbial populations do not change (Dell’Anno et al., 1998; Bodrossy et al., 2006). Also, RNA-based analyses would be influenced greatly by environmental conditions when sampling, while DNA-based analyses could include “historical” information (Anderson and Parkin, 2007) from inactive (e.g., dormant cells and spores) and dead cells (Dell’Anno et al., 1998; Bodrossy et al., 2006).

Stimulated gene expressions for degrading more recalcitrant C components under warming were likely linked to the quality decrease in soil C input due to the plant community shift toward more C_4_ species. Consistently, significant correlations were observed between expressed genes for degrading more recalcitrant C (e.g., aromatic components) and the measured soil recalcitrant C pool. In this experimental site, the AGB increase likely brought more labile C components into the soil and the microorganisms with higher capacity of degrading recalcitrant C components may not gain fitness in the community structure in the long-term as reflected by the DNA analysis. However, the metabolic state of those microorganisms could be possibly changed as reflected by corresponding expressed gene transcripts. Disproportional expressions of genes to the abundances of their originated cells were also observed by Baldrian et al. (2012) who found that some of the most abundant expressed *cbhI* genes for cellulose hydrolysis were from low abundance fungi species. Microorganisms have the ability to mediate their metabolic states in different niches, which are governed by the principles of redox reactions and chemical thermodynamics (Merlo et al., 2011). It has been reported that the soil substrate availability (poor or rich in organic C) could affect the metabolic enthalpy change of microbial activity (Barros et al., 2000). The stimulated expressions of genes involved in more recalcitrant C degradation may decrease the long-term soil C stability and trigger a positive feedback to climate warming.

Different changing directions (increase and decrease) for expressed genes involved in labile C degradation and N cycling may partially or completely reflect the dynamics of labile C components and available N at the time of sampling, which may mask the general trends of warming influences as reflected by the DNA analysis. Both labile C and available N pools have short mean residence time (MRT) in soils. The MRT of active fraction of soil organic C (e.g., plant residues and metabolites from soil biota) is only in months (Paul et al., 2001). Though the MRT of plant N in soils is in years (Silla and Escudero, 2004; Finzi et al., 2007), the available N (i.e., NH_4_^+^ or NO_3_^-^) could be transferred or taken up by plants and microorganisms in a much shorter time, like days or even hours (Inselsbacher et al., 2010). These facts imply that these pools, especially the available N, may be quite dynamic in the field, thereby likely resulting in different changing directions (increase and decrease) of corresponding gene transcripts.

Soil moisture, a dynamic variable in the field along time, was important in shaping expressed functional genes according to its significant correlations with the structures of C degradation or N genes. Interestingly, these expressed genes were also correlated with a few more “stable” variables. For example, the structure of all expressed C degradation genes were correlated with the TOC or C content in C_4_ species leafs; while the structure of all expressed N genes were correlated with the N content or C:N ratio in C_4_ species leafs. Linkages of RNA-based community composition with both dynamic and stable environmental attributes imply that information obtained from RNA analyses was still capable of capturing some stable trends caused by the long-term treatment, though DNA analyses would be better for this purpose. It may depend on both the strength of treatment effects and the dynamics of changes.

P and S are critical nutrient elements for plant growth (Eaton, 1950; Stewart et al., 1966; Fredeen et al., 1989). In P and S cycling, the average abundances of expressed genes were all higher under warming, suggesting a stable trend of accelerated nutrient cycling processes. This phenomenon was possibly related to the enhanced substrate input from plants, as well as promoted uptake from biological communities. The photosynthetic P use efficiency was suggested to be similar in C_3_ and C_4_ species (Halsted and Lynch, 1996). The P demand could be higher along with the stimulated growth of C_4_ species under warming. The S assimilatory pathway in plants is known to be well-coordinated with the N assimilatory pathway (Reuveny et al., 1980; Koprivova et al., 2000; Prosser et al., 2001; Kopriva and Koprivova, 2005) and the plant N use efficiency could be limited by the deficiency of soil S supply (Salvagiotti et al., 2009). In this field site, the N use efficiency was higher in C_4_ species and increased by warming (Brown, 1978; Oaks, 1994; An et al., 2005), which may demand higher S supply. In this way, if the accelerated P and S cycling mediated by microorganisms led to higher supplies of P and S, it was likely to serve as a key mechanism to sustain higher plant growth in the long-term warming treatment.

**FIGURE 6:**
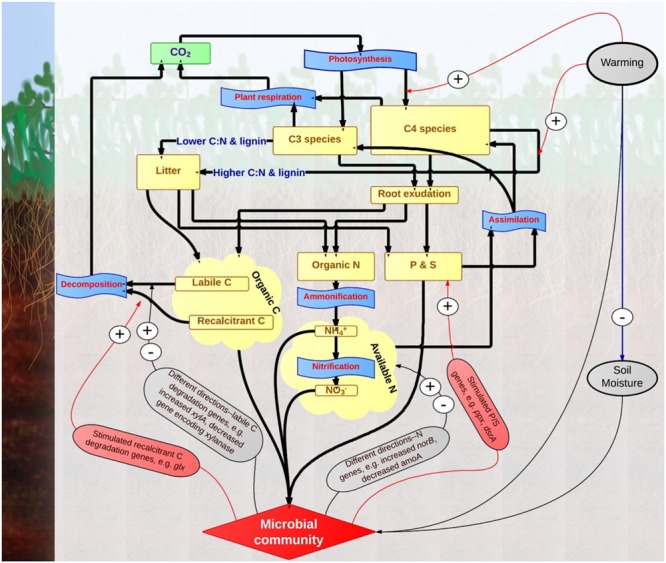
**Conceptual model of warming impacts on grassland ecosystem processes based on results from this study.** Greenhouse gas pool of CO_2_ is represented by square frames in green color, material pools are represented by square frames in yellow color, and biological processes are represented by punched tape frames in blue color. Material flows are represented by thicker rows in black color. Impacts of environmental attributes (e.g., soil temperature) and microbial community are represented by narrow rows in red (increase), blue (decrease) or black (different directions) colors.

In summary, substantial changes of expressed functional genes in soil microbial communities were observed in this experimental site after long-term warming treatment. In the previous study, shifts in the functional gene composition of soil microbial community, enriched genes for labile C but not recalcitrant C degradation, and enriched genes for nutrient cycling were discovered under warming based on DNA-GeoChip analysis (Zhou et al., 2012). Findings from this study based on RNA-GeoChip analysis were illustrated in a conceptual model (**Figure 6**). Warming stimulated the growth of C_4_ species with higher C:N ratio and possibly greater lignin content. Thus, the quality of soil C input from plant decreased under warming, likely linked to stimulated gene expressions for degrading more recalcitrant C. Moreover, consistent trends of stimulated expressions were observed for genes in phosphorus and sulfur cycling, but not in labile carbon degradation and nitrogen cycling. These results suggest that the RNA-based GeoChip analysis is a robust tool to analyze soil microbial community in global climate change studies, though its combination with DNA-based analysis is needed as the dynamics of gene expression at the time of sampling may mask the general trends that can be captured by the DNA analysis. The stimulated genes expressions for degrading recalcitrant C may reveal changes in metabolic states of soil microorganisms with pathways of degrading recalcitrant C, likely to decrease the long-term soil C stability and trigger a positive feedback to climate warming. However, the accelerated P and S cycling processes implied by strong stimulating impacts of warming on P and S gene expressions may serve as a key negative feedback mechanism to sustain higher plant growth under warming. Though further studies are still needed to evaluate the weight of each mechanism in determining the whole direction of ecosystem feedback, our findings elucidate the importance of soil microbial community in regulating ecosystem responses to climate warming.

## Author Contributions

All authors contributed intellectual input and assistance to this study and manuscript preparation. JZ and YL developed the original concepts. KX, JX, AZ, FL, DL, LW, YD, ZH, and JN contributed reagents, experiment conduction, data collection, and data analysis. Specifically, JX, AZ, KX, and FL handled all soils processing and subsampling for microbial analysis. JX and AZ did GeoChip hybridization. KX, YD, LW, ZH, and JN performed data analysis. KX wrote the manuscript with help from JZ, YL, FL, LW, ZH, and JN. All authors involved in revising this manuscript.

## Conflict of Interest Statement

The authors declare that the research was conducted in the absence of any commercial or financial relationships that could be construed as a potential conflict of interest.

## Footnotes

1 http://ieg.ou.edu/microarray/
