# Supplementary material for: Warming Alters Expressions of Microbial Functional Genes Important to Ecosystem Functioning
Source: Front Microbiol. 2016 May 6;7:668. doi: 10.3389/fmicb.2016.00668 (PMC4858606; doi:10.3389/fmicb.2016.00668)
Supplement: Supplementary file 1 [file Table_1.DOCX]

**Supplementary Table**

Table S1. Environmental attributes measured in 2008 for warming and control samples (mean ± standard error). Significance was tested by paired two-tailed t test and the significance was labelled with ** when p<0.05, and * when p<0.10. Environmental attributes from previous studies were labeled with their data source.

|  | Variable | Control | Warming |  | Source |
| --- | --- | --- | --- | --- | --- |
| Plant biomass | C_3_ aboveground biomass (g m^-2^) | 88.00±14.80 | 81.05±4.90 |  | Xu et al., 2012a |
|  | C_4_ aboveground biomass (g m^-2^) | 188.99±13.98 | 301.39±35.98 | ** | Xu et al., 2012a |
|  | Total aboveground biomass (g m^-2^) | 276.99±3.23 | 382.44±33.22 | ** | Xu et al., 2012a |
|  | C_4_:C_3_ in aboveground biomass | 2.47±0.40 | 3.85±0.53 | ** | Xu et al., 2012a |
|  | ANPP (g m^-2^) | 590.00±15.23 | 611.18±57.95 |  | Xu et al., 2014 |
|  | BNPP (g m^-2^) | 321.88±30.05 | 404.83±37.91 |  | Xu et al., 2014 |
| Plant leaf | C_3_-C content (%) | 44.20±0.78 | 43.48±0.64 |  | Niu et al., 2010 |
|  | C_3_-N content (%) | 1.43±0.19 | 1.27±0.13 |  | Niu et al., 2010 |
|  | C_3_-C:N | 33.48±4.09 | 36.43±4.25 |  | Niu et al., 2010 |
|  | C_4_-C content (%) | 44.59±0.13 | 45.31±0.47 | * | Niu et al., 2010 |
|  | C_4_-N content (%) | 0.77±0.03 | 0.70±0.06 | * | Niu et al., 2010 |
|  | C_4_-C:N | 58.88±2.95 | 67.05±5.78 | ** | Niu et al., 2010 |
| Soil Microclimate | Annual Temperature (°C) | 16.82±0.19 | 18.02±0.29 | ** |  |
|  | Annual Moisture (%) | 26.96±0.70 | 25.21±0.64 | ** | Xu et al., 2012b |
| Soil C and N | Labile C pool 1 (mg C g^-1^ dry soil) | 2.77±0.42 | 2.75±0.33 |  | Zhou et al., 2012 |
|  | Labile C pool 2 (mg C g^-1^ dry soil) | 3.84±0.51 | 4.34±0.58 |  | Zhou et al., 2012 |
|  | Recalcitrant C pool (mg C g^-1^ dry soil) | 7.09±0.75 | 7.17±1.62 |  | Zhou et al., 2012 |
|  | Total organic C (mg C g^-1^ dry soil) | 13.71±1.01 | 14.26±2.01 |  | Zhou et al., 2012 |
|  | δ^13^C (‰) | -18.48±0.53 | -17.26±0.76 | ** | Zhou et al., 2012 |
|  | Soil C derived from C4 species (%) | 60.2±3.5 | 71.5±5.2 | ** | Cheng et al., 2011 |
|  | NH_4_^+^ (mg N g^-1^ dry soil) | 7.90±0.92 | 9.01±2.84 |  |  |
|  | NO_3_^-^ (mg N g^-1^ dry soil) | 5.37±1.96 | 4.39±0.96 |  |  |
|  | Total N (%) | 0.11±0.01 | 0.12±0.01 |  |  |
|  | δ^15^N | 2.66±0.30 | 3.09±0.21 |  | Zhou et al., 2012 |
| Annual Ecosystem C flux | Soil respiration (g CO_2_-C m^-2^ day^-1^) | 2.32±0.21 | 2.75±0.32 |  | Xu et al., 2015 |
|  | Heterotrophic respiration (g CO_2_-C m^-2^ day^-1^) | 1.24±0.19 | 1.61±0.22 | * |  |
|  | Autotrophic respiration (g CO_2_-C m^-2^ day^-1^) | 1.08±0.23 | 1.14±0.24 |  |  |

**Supplementary Materials and Methods for Environmental Attributes**

1. **Soil temperature**

Soil temperature was measured every ten minutes by thermocouples installed at the 2.5cm depth at subplot centers, which were connected to a CR10 datalogger (Campbell Scientific Inc., Utah, US). The averages of soil temperature for every hour were stored in an SM196 Storage Module (Campbell Scientific Inc., Utah, US). However, technical problems occurred in the field for the datalogger to collect data in some periods of 2008 in two control plots and one warming treatment plot. To avoid missing data that is not allowed for CCA and Mantel test, we selected the dataset of soil temperature during days when soil moisture, soil respiration and heterotrophic respiration were measured together, once or twice a month, to calculate the yearly average.

1. **Soil Analyses**

For soil samples collected in October, 2008, soil NH_4_^+^ and NO_3_^-^ contents were extracted by 1M KCl and measured by Lachat Quickchem 8500 series 2 (Lachat, Loveland, CO), and the total soil N content was determined by LECO TruSpec (LECO Corporation, St. Joseph, MI) in the Soil, Water and Forage Analytical Laboratory at the Oklahoma State University (Stillwater, OK).

1. **Heterotrophic and autotrophic respiration measurements**

A LI-COR 6400 portable photosynthesis system attached to a soil CO_2_ flux chamber (LI-COR Inc., Lincoln, NE, USA) was used to measure the soil respiration once or twice a month between 10:00 and 15:00 (local time). The CO_2_ flux were measured above a PVC collar (80 cm^2^ in area and 5 cm in depth) and a PVC tube (80 cm^2^ in area and 70 cm in depth) in each plot. Before measurements, aboveground living plants were taken out of PVC tubes and collars every time. PVC tubes prevented new root from growing inside the tubes. Thus, the CO2 efflux measured above PVC tubes represented the heterotrophic respiration; whilst the CO2 efflux measured above PVC collars represented the total soil respiration including both heterotrophic and autotrophic respiration. The autotrophic respiration was calculated as the difference between measurements above PVC collars and tubes. Soil respiration and heterotrophic respiration were measured once or twice a month. The yearly averages of heterotrophic respiration and autotrophic respiration in 2008 were presented in this study.

**Reference**

Cheng, X., Luo, Y., Xu, X., Sherry, R., and Zhang, Q. (2011). Soil organic matter dynamics in a North America tallgrass prairie after 9 yr of experimental warming. *Biogeosciences* 8**,** 1487-1498.

Niu, S.L., Sherry, R.A., Zhou, X.H., Wan, S.Q., and Luo, Y.Q. (2010). Nitrogen regulation of the climate-carbon feedback: evidence from a long-term global change experiment. *Ecology* 91, 3261–3273

Xu, X., Sherry, R.A., Niu, S.L., Zhou, J.Z., and Luo, Y.Q. (2012a). Long-term experimental warming decreased labile soil organic carbon in a tallgrass prairie. *Plant Soil* 361: 307-365, doi: 10.1007/s11104-012-1265-9.

Xu, X., Niu, S.L., Sherry, R.A., Zhou, X.H., Zhou, J.Z., and Luo, Y.Q. (2012b). Interannual variability in responses of belowground NPP and NPP partitioning to long-term warming and clipping in a tallgrass prairie. *Global Change Biol.* 18, 1648–1656.

Xu, X., Luo, Y.Q., Shi, Z., Zhou, X.H., and Li, D.J. (2014). Consistent proportional increments in responses of belowground net primary productivity to long-term warming and clipping at various soil depths in a tall-grass prairie. *Oecologia* 174 (3), 1045-1054.

Xu, X., Shi, Z., Li, D.J., Zhou, X.H., Sherry, R.A., and Luo, Y.Q. (2015). Plant community structure regulates responses of prairie soil respiration to decadal experimental warming. *Global Change Biol.* 21(10), 3846-3853.

Zhou, J.Z., Xue, K., Xie, J.P., Deng, Y., Wu, L.Y., Cheng, X.H., Fei, S.F., Deng, S.P., He, Z.L., Van Nostrand, J.D., and Luo, Y.Q. (2012). Microbial mediation of carbon-cycle feedbacks to climate warming. *Nat. Clim. Change* 2**,** 106-110. doi: Doi 10.1038/Nclimate1331.
